# Management of a Submacular Hemorrhage Secondary to Age-Related Macular Degeneration: A Comparison of Three Treatment Modalities

**DOI:** 10.3390/jcm9103088

**Published:** 2020-09-24

**Authors:** Seongyong Jeong, Dong-Geun Park, Min Sagong

**Affiliations:** 1 Department of Ophthalmology, Yeungnam University College of Medicine, Daegu 42415, Korea; jjsssyyyy@naver.com (S.J.); bluepdg@naver.com (D.-G.P.); 2 Yeungnam Eye Center, Yeungnam University Hospital, Daegu 42415, Korea

**Keywords:** age-related macular degeneration, anti-VEGF, pneumatic displacement, submacular hemorrhage, tissue plasminogen activator

## Abstract

This paper aims to compare the effects of three treatment modalities for a submacular hemorrhage (SMH) secondary to exudative age-related macular degeneration (AMD). Seventy-seven patients with an SMH were divided into three groups: small-sized (optic disc diameter (ODD) ≥ 1 to < 4), medium-sized (ODD ≥ 4 within the temporal arcade) and large-sized (ODD ≥ 4, exceeding the temporal arcade). Patients received anti-vascular endothelial growth factor (anti-VEGF) monotherapy, pneumatic displacement (PD) with anti-VEGF or a vitrectomy with a subretinal tissue plasminogen activator (tPA) and gas tamponade based on the surgeon’s discretion. The functional and anatomical outcomes were evaluated. Among the 77 eyes, 45 eyes had a small-sized, 21 eyes had a medium-sized and 11 eyes had a large-sized SMH. In the small-sized group, all treatment modalities showed a gradual best-corrected visual acuity (BCVA) improvement with high hemorrhagic regression or displacement rates (over 75%). In the medium-sized group, PD and surgery were associated with better BCVA with more displacement than anti-VEGF monotherapy (67% and 83%, respectively, vs. 33%). In the large-sized group, surgery showed a better visual improvement with a higher displacement rate than PD (86% vs. 25%). Our findings demonstrated that visual improvement can be expected through appropriate treatment strategy regardless of the SMH size. In cases with a larger SMH, invasive techniques including PD or surgery were more advantageous than anti-VEGF monotherapy.

## 1. Introduction

Submacular hemorrhage (SMH) is one of the severe complications in patients with neovascular age-related macular degeneration (AMD). As it physically separates the neurosensory retina from the retinal pigment epithelium (RPE) layer, the resulting barrier effect interferes with the diffusion of nutrients and metabolites [1]. Additionally, the shearing of the photoreceptor from enmeshed fibrin and iron toxicity in the retina due to the blood cause photoreceptor death leading to a poor visual prognosis [1,2].

Various treatment modalities for SMH have been developed. Expansile gas injection including perfluoropropane (C_3_F_8_) or sulfahexafluoride (SF_6_) can be used to push away the SMH from the fovea. A tissue plasminogen activator (tPA), a serine protease, dissolves the blood clot by cleaving the fibrin [3]. Anti-vascular endothelial growth factor (anti-VEGF) drugs affect the underlying choroidal neovascularization (CNV) and treat the SMH [4]. A surgical intervention, pars plana vitrectomy (PPV), facilitates vitreous oxygenation and reduces the VEGF level [5]. Single or combined modalities have been used to treat the SMH.

A widely used treatment option for the SMH is anti-VEGF monotherapy. It has shown favorable results but severe cases were often excluded from previous studies [6,7,8,9]. Thick blood under the fovea can be displaced by the tPA and subsequent expansile gas. However, when patients received high concentrations of the tPA, i.e., over 100 μg, retinal toxicity could occur [10]; its limited efficacy has been reported in previous studies [11,12]. A retinal tear or detachment could be observed after intravitreal gas injection [13]. Moreover, there are cases in which a tPA with gas injection should be avoided such as in patients with superior-juxtafoveal SMH and those who cannot maintain a prone position [14]. Although surgical treatment with PPV, a subretinal tPA and expansile gas have been reported to provide better visual acuity, the procedure is more complex and needs additional special instruments such as a 41-gauge cannula. Therefore, an appropriate treatment selection per case may be essential for patients with an SMH. To date, the rarity of the disease [15] and devasting prognosis [16] have limited randomized clinical trials and there is a lack of consensus on the standard treatment of patients with AMD complicated by an SMH.

Hence, we sought to compare the anatomical and functional outcomes of practically selected treatment modalities depending on the hemorrhage size in patients with AMD complicated by an SMH. We also evaluated the possible prognostic factors for visual outcomes.

## 2. Materials and Methods

A retrospective chart review of patients with an SMH from March 2015 to November 2018 at Yeungnam University Hospital was performed. The Institutional Review Board of the Yeungnam University Hospital approved the protocol. Data were collected from medical charts of patients who had an SMH involving the fovea secondary to exudative AMD. Additional inclusion criteria were age > 50 years and SMH thickness > 250 μm.

The baseline evaluation included best-corrected visual acuity (BCVA) with a Snellen chart, dilated fundus examination, fundus photography and spectral domain optical coherence tomography (SD-OCT). An angiography with fluorescein or indocyanine green was performed to identify the underlying lesion.

The fundus images were evaluated with ImageJ software (National Institutes of Health, Bethesda, MD) for Windows. The longest diameter of the SMH was measured and recorded as multiple values of the optic disc diameter (ODD). Patients were divided into three groups based on the size of the SMH: small-sized (≥ 1 to < 4-disc ODD), medium-sized (≥ 4 ODD and not extending beyond the temporal arcade) and large-sized (≥ 4 ODD and extending beyond the temporal arcade) [5]. The different treatment outcomes among the subgroups were compared.

Using SD-OCT (Spectralis; Heidelberg Engineering, Heidelberg, Germany), a standard volume scan consisting of a 6 × 6 mm area was performed at the macular area and at points considered to have the largest SMH. A retinal specialist (Dong-Geun Park) reviewed all the images and measured the maximum height of the SMH using a manual ruler in the integrated software.

Patients were prescribed a treatment modality based on the surgeon’s judgement of optimal treatment. Twenty-nine patients were treated with an intravitreal anti-VEGF monotherapy (bevacizumab, ranibizumab or aflibercept). Twenty-five patients received pneumatic displacement (PD) using 0.3 cc of 100% C_3_F_8_ gas with an intravitreal anti-VEGF injection simultaneously with or without an intravitreal tPA. When anti-VEGF therapy was combined with the tPA, aflibercept, which is known to be cleaved by the tPA [17], was avoided. Twenty-three patients underwent a 25-gauge PPV followed by a subretinal tPA (Actilyse, Boehringer Ingelheim, Germany) injection with a 41-gauge flexible needle (De Juan/Awh Subretinal Injection Cannula, Synergetics Inc, USA) and a 20% SF_6_ gas filling through a fluid–gas exchange.

The surgeon applied these three strategies according to the SMH size. Anti-VEGF monotherapy was considered for the small-sized SMH preferentially. C_3_F_8_ gas with an anti-VEGF injection (C_3_F_8_/anti-VEGF) was selected mainly for the medium-sized SMH and a PPV with a subretinal tPA and SF_6_ gas (PPV/tPA/ SF_6_) was preferred for the large-sized SMH, respectively. Additionally, parameters including location, thickness and duration of the hemorrhage were considered to achieve the best outcomes for each patient when choosing the treatment option. Every procedure was performed by a retinal surgeon (Min Sagong). Patients who received any gas injections were instructed to maintain a face-down position for 2–3 days.

If a patient complained of decreased vision during the follow-up period, we performed thorough examinations to detect any sign of recurrence including a newly developed SMH, a small retinal hemorrhage, increased retinal thickness on an OCT, leakage on an angiography or decreased vision alone. Based on the surgeon’s decision, additional anti-VEGF injections were administered.

The main outcomes were the degree of displacement or regression of the SMH from the fovea at 1 month after treatment; BCVA at 1, 3, 6 and 12 months after the initial treatment; prognostic factors for visual acuity; frequency of additional anti-VEGF injections; incidence of a recurrent SMH or breakthrough vitreous hemorrhage and other complications.

Statistical analyses were performed using IBM SPSS ver. 20.0 for Windows (IBM Co., Armonk, New York, USA). BCVA was measured using a Snellen chart and converted to the logarithm of the minimum angle of resolution (logMAR) units for statistical analyses. An analysis of variance or a Kruskal–Wallis test was performed to determine the differences in the baseline parameters among the subgroups. If there was a difference, a post-hoc analysis with a Bonferroni correction was performed to identify the groups that caused that significance. Categorical variables among the subgroups or among the treatment modalities were analyzed by χ2 test or Fisher’s exact test. To identify factors that correlated with BCVA at 3 months and 12 months, a Pearson correlation test and analysis of covariance (ANCOVA) were performed. Variables with a *p*-value < 0.05 were considered significant.

## 3. Results

### 3.1. Baseline Characteristics

Among the 77 eyes of 77 patients with hemorrhagic AMD, 45 eyes had a small-sized, 21 eyes had a medium-sized and 11 eyes had a large-sized SMH. The baseline characteristics of the affected eyes are provided in Table 1. Eyes in three groups did not show a difference in terms of age (*p* = 0.164), sex (*p* = 0.755), duration of symptoms (*p* = 0.653), lens status (*p* = 0.119) and proportion of anticoagulant usage (*p* = 0.068). The mean size of the SMH as an ODD was 2.45 ± 0.90, 5.09 ± 1.27 and 7.55 ± 2.27 in the small-, medium- and large-sized subgroups (*p* < 0.001), respectively. The mean SMH thickness in each group increased according to the SMH size (474.6 ± 149.6, 791.9 ± 482.0 and 955.4 ± 375.9 µm, respectively; *p* < 0.001). Regarding the AMD lesions, the small-sized group showed a higher percentage of polypoidal choroidal vasculopathy (PCV) (66.7%) than CNV (33.3%). In contrast, the large-sized group consisted of more CNV (72.7%) than PCV (27.3%). There was a statistical difference in the distribution of classifications among the three groups (*p* = 0.028). The treatment modalities were differently distributed in each subgroup (*p* = 0.011). Anti-VEGF monotherapy was applied mainly in the small-sized group (51.1%) and also applied in the medium-sized (28.6%) groups. A combination of PD with anti-VEGF and/or an intravitreal tPA injection was applied in all subgroups (26.7% in the small-, 42.8% in the medium- and 36.4% in the large-sized groups). A PPV with a subretinal tPA and SF_6_ gas, although also applied in the small- (22.2%) and medium-sized (28.6%) groups, was mainly applied in the large-sized group (63.6%).

### 3.2. SMH Displacement or Regression Rate

The distribution of the SMH displacement did not significantly differ among the subgroups (*p* = 0.200; Table 2). All three groups achieved a relatively high percentage of complete SMH displacement or regression (82%, 62% and 73%, respectively); however, it was achieved by different modalities in each group. In the small-sized group, anti-VEGF monotherapy achieved 78% of the complete SMH regression and C_3_F_8_/anti-VEGF and a PPV/tPA/SF_6_ showed 75% and 100% of complete displacement, respectively. In the medium-sized group, anti-VEGF monotherapy showed poor anatomical outcomes (33% of complete regression) while the other two treatment methods showed acceptable results (67% and 83% of complete displacement, respectively). In the large-sized group, the PPV/tPA/SF_6_ contributed to the high percentage of complete displacement (86%) but C_3_F_8_/anti-VEGF did not (25%) (Table 2).

### 3.3. Visual Outcomes

The baseline mean BCVA differed among the groups (*p* = 0.015). The post-hoc analysis revealed a significant effect of the large-sized group when compared with that of the small-sized group (*p* = 0.014). The overall mean logMAR BCVA improved from 1.30 ± 0.83 at baseline to 1.03 ± 0.95 at 3 months (*p* = 0.040), 1.00 ± 0.93 at 6 months (*p* = 0.044) and 0.76 ± 0.83 at 12 months (*p* = 0.008). Each subgroup showed an improvement of mean BCVA at each follow-up; the difference in the baseline mean BCVA among the subgroups disappeared at 6 months (*p* = 0.145) and 12 months (*p* = 0.139) (Table 2).

**Figure 1:**
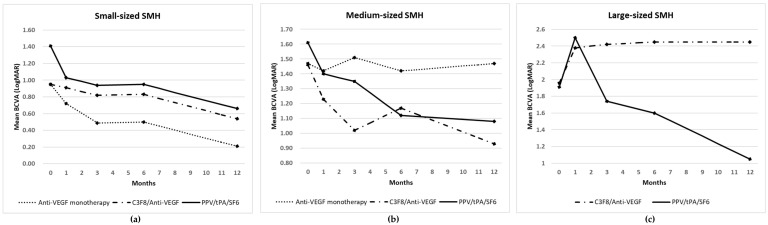
Changes in the mean best-corrected visual acuity (BCVA) in the (**a**) small-, (**b**) medium- and (**c**) large-sized submacular hemorrhage (SMH) groups. The eyes were treated with anti-vascular endothelial growth factor (anti-VEGF) monotherapy, pneumatic displacement (PD) using perfluoropropane (C_3_F_8_) with an anti-VEGF injection or a pars plana vitrectomy (PPV) with a subretinal tissue plasminogen activator (tPA) and gas tamponade using sulfahexafluoride (SF_6_). C_3_F_8_/Anti-VEGF, PD with anti-VEGF; LogMAR, logarithm of the minimal angle of resolution; PPV/tPA/SF_6_, PPV with subretinal tPA and gas tamponade.

In the subgroup analyses, the small-sized group showed an improved mean BCVA regardless of the treatment modality. In the medium-sized group, anti-VEGF monotherapy had little effect on the visual improvement while the other modalities improved the mean BCVA for 12 months. In the large-sized group, the surgeon excluded anti-VEGF monotherapy. C_3_F_8_/anti-VEGF was used in 36.4% and a PPV/tPA/SF_6_ was employed in 63.6% of patients. The mean BCVA improved only in the PPV/tPA/SF_6_ group and decreased in the C_3_F_8_/anti-VEGF group (Table 3; Figure 1).

### 3.4. Factors Associated with Visual Outcomes

**Figure 2:**
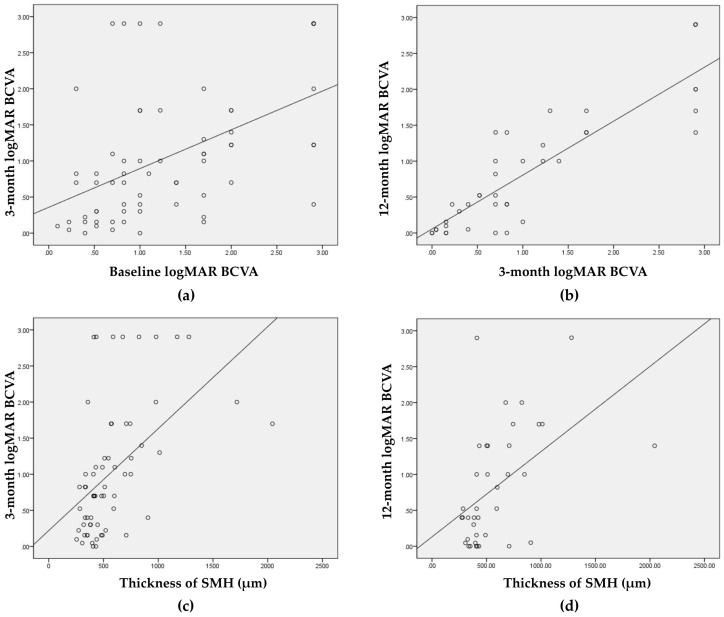
Scatter plots comparing (**a**) baseline BCVA and postoperative BCVA at 3 months and (**b**) BCVA at 3 months and at 12 months, as well as SMH thickness and (**c**) BCVA at 3 months and (**d**) BCVA at 12 months. BCVA, best-corrected visual acuity; LogMAR, logarithm of the minimum angle of resolution; SMH, submacular hemorrhage.

A significant positive correlation was identified between the BCVA at baseline and 3 months (*r* = 0.543, *p* < 0.001) and between the BCVA at 3 months and 12 months (*r* = 0.875, *p* < 0.001). The SMH thickness also showed a positive correlation with BCVA at 3 months (*r* = 0.592, *p* < 0.001) and 12 months (*r* = 0.497, *p* = 0.001; Figure 2).

To identify the effect of treatment modality on visual acuity during the follow-up, ANCOVA with baseline visual acuity as a covariance was performed, which is a known prognostic factor. However, different treatment modalities did not influence the visual outcomes. We divided patients into two groups based on the SMH duration (duration ≤ 14 days or > 14 days) and performed ANCOVA with baseline visual acuity as a covariant. There was a significant difference in visual outcomes (*p* = 0.022 at 3 months and *p* = 0.022 at 12 months) between the two groups.

### 3.5. Frequency of Additional Anti-VEGF Injections

For 12 months, additional anti-VEGF injections were administered to 66.7%, 71.4% and 72.7% of eyes in each group (Table 2). There was no statistical significance (*p* = 0.886). The mean number of injections were 3.27 ± 2.06, 2.60 ± 1.45 and 3.00 ± 1.85 during 12 months in each group (*p* = 0.526).

### 3.6. Complications

Breakthrough vitreous hemorrhage occurred in three (6.7%), one (4.8%) and four (36.4%) eyes of each group. One case of a recurrent SMH, one case of an RPE rip and one case of a macular hole were observed in the medium-sized group. (Table 2). The RPE rip and macular hole were associated with the PPV/tPA/SF_6_.

## 4. Discussion

In this study, we compared three treatment modalities for AMD complicated by an SMH. We included an extended spectrum of hemorrhage sizes as well as a larger number of patients than previous studies. Moreover, we divided our cases according to the SMH size and analyzed the treatment outcomes of three different modalities to identify their effects in each subgroup with homogeneity in terms of size.

The overall mean BCVA improved significantly from the baseline during the 12 month follow-up period. However, different outcomes were observed according to the SMH size and treatment modalities. In the small-sized group, anti-VEGF monotherapy was performed on the majority of patients (51.1%) and it significantly improved the visual acuity. The two other treatment modalities also increased the mean BCVA and the magnitude of improvement showed no difference among the treatment modalities. This result may be related to the anatomical outcome that a small-sized SMH was effectively regressed by anti-VEGF only (75% of complete regression) without gas or vitrectomy. Consistent with our results, previous studies reported the efficacy of anti-VEGF monotherapy with a minimally invasive technique [6,7,9,18,19]. This result suggests that a small-sized SMH could be resolved with anti-VEGF only, even in the absence of additional treatments accelerating the displacement of the hemorrhage.

However, anti-VEGF monotherapy was no longer effective in the medium-sized group while the other two modalities increased the mean BCVA. Moreover, anatomical outcomes were worse with monotherapy (33% of complete regression) than with other modalities (67% and 83% of complete displacement, respectively). In a retrospective study comparing anti-VEGF monotherapy with a combination of anti-VEGF, tPA and gas [19], the combination therapy groups showed better outcomes than the monotherapy group (mean changes of + 1 Snellen line vs. 0 line). A more recent study comparing ranibizumab monotherapy with a combination of PD with ranibizumab [20] reported that a combination therapy with a higher proportion of 0.3 lines or more visual improvement tended to be more effective than monotherapy (57.1% vs. 37.9%) although mean changes in BCVA showed no statistical difference between groups. Thinner and smaller hemorrhages were reported in their study than in our study (269 µm vs. 792 µm in the medium-sized group and 8.2 disc area [DA] vs. 15.3–20.3 DA in the medium-sized group when the SMH area was recalculated from the ODD) and might cause no significant difference between the treatment modalities. Shin et al. [21] compared the combination of PD with anti-VEGF and anti-VEGF monotherapy. Although both groups achieved a significant improvement in BCVA at 6 months, the short-term visual outcomes at 1 month were better in the combination group than in the monotherapy group, suggesting different regression rates between the groups. Their SMH size was also smaller than that in our study (9.3 DA when it was recalculated vs. 15.3–20.3 DA). Thick and large SMHs may not be absorbed efficiently with anti-VEGF only, which limits the visual improvement and requires additional treatment options to displace the hemorrhage from the fovea.

In this study, the only effective treatment option for visual improvement in the large-sized SMH was the PPV/tPA/SF_6_. Limited studies have reported the efficacy of a vitrectomy and subretinal tPA for SMH [5]. Fassbender et al. reported that the PPV/tPA/SF_6_ showed a better outcome than PD only (changes of mean BCVA were −0.90 vs. +0.10 logMAR units) [22]. Treumer et al. treated SMH patients with a PPV, a subretinal tPA and anti-VEGF [23]. The mean SMH size was 4.3 ODD (range 1.5–15) and the mean thickness was 762 µm (range 217–1840 µm), similar to those in our study. They reported complete displacement of the SMH in 87% of eyes. In this study, the high percentage of complete displacement in the large-sized group was achieved with a PPV/tPA/SF_6_ (86%) but not with C_3_F_8_/anti-VEGF (25%). This result suggests that for a massive SMH, a vitrectomy with a subretinal tPA is more efficient than PD, showing better functional and anatomical outcomes.

The amount of the SMH that has been considered a prognostic factor for visual outcomes [23] is clinically measured in terms of thickness or diameter. The duration of the SMH is also a prognostic factor in a SMH [24] although several studies have reported contrasting results [11,25]. In this study, we found a positive correlation between the SMH thickness and visual outcomes. Additionally, eyes with a hemorrhage duration ≤ 14 days showed better visual outcomes than those with a duration > 14 days. Thus, the rapid removal of a hemorrhage within 14 days is important for patients with a massive SMH.

In this study, a strong correlation of BCVA at 3 months with the BCVA at 12 months was identified, suggesting that optimal initial treatment and subsequent anti-VEGF retreatment may be helpful to maintain the initial visual gain. Treumer et al. [23] reported a stronger positive correlation between the 3 month and final BCVA than between the baseline and final BCVA, similar to that in our study. They adopted strict retreatment regimens with anti-VEGF based on OCT and functional criteria [26]. After the initial treatment, we also administered additional anti-VEGFs as needed based on OCT findings, which may have contributed to the maintenance of visual acuity.

Of the complications during the follow-up, vitreous hemorrhage was more common in the large-sized group (36.4%) than in other groups (6.7% and 4.8%, respectively). However, the percentage of vitreous hemorrhage was not different among the treatment modalities (10%, 12% and 9%, respectively). Although the PPV/tPA/SF_6_ was a more invasive technique, intraoperative and other postoperative complications were rarely observed. One case of an RPE rip (4.3%), one of a macular hole (4.3%) and none of retinal detachment (RD) were observed, similar to that in previous studies (3.4~12% of RPE rip, 2.4~4.2% of macular hole and 2.4~8.3% of RD) [27,28,29]. This may be partially attributable to the small number of patients treated with a PPV/tPA/SF_6_. All procedures were done by an experienced surgeon, which minimized complications. It was also helpful to try to find a suitable location for the subretinal injection location while avoiding large blood vessels and pigment epithelial detachment using multimodal images including fundus photography and OCT. The rarity of complications associated with the PPV/tPA/SF_6_ and its efficacy in the large-sized hemorrhage group could justify our treatment selection.

Our study has several limitations. Due to the retrospective characteristics, the treatment modalities were not consistently applied in each group according to the SMH size, resulting selection bias. However, all judgments were made by one experienced surgeon to minimize bias in the choice of treatment options. In addition, due to the nature of the disease, the number of patients with a large SMT was small, which limited the statistical analysis. The scarcity of the disease and the fact that inadequate treatment in time can lead to severe vision loss make it difficult to conduct large-scale prospective studies for a limited period along with ethical issues. Therefore, in the study of rare diseases, the real-world data including many patients can be more important.

In conclusion, the individualized treatment options for a SMH depending on the hemorrhage size showed acceptable anatomical outcomes with visual improvement in each subgroup. For the small-sized SMH, anti-VEGF monotherapy was useful. However, in the medium-sized SMH, anti-VEGF only was not effective and the rapid removal of the hemorrhage was required for a thick SMH. For a large-sized SMH, a vitrectomy with a subretinal tPA and expansile gas were the only effective treatment options. Our strategy of selecting the treatment modality according to the hemorrhage size could be a reference for the treatment of SMHs.

## Figures and Tables

**Table 1:** Baseline Characteristics.

	Small-Sized SMH	Medium-Sized SMH	Large-Size dSMH	*p*-Value	Total
No. of patients	45	21	11		77
Mean age, years	71.5 ± 10.9	74.8 ± 11.1	72.3 ± 9.0	0.164 ^a^	73.2 ± 10.8
Sex (%)				0.755 ^b^	
Male	33 (73.3)	14 (66.7)	7 (63.6)		54 (70.1)
Female	12 (26.7)	7 (33.3)	4 (36.4)		23 (29.9)
Systemic anticoagulants (%)	9 (20.0)	10 (47.6)	3 (27.4)	0.068 ^b^	22 (100.0)
Duration of SMH, days	16.6 ± 30.6	10.6 ± 19.7	12.4 ± 11.9	0.653 ^a^	14.3 ± 25.8
Mean size of SMH, ODD	2.45 ± 0.90	5.09 ± 1.27	7.55 ± 2.27	<0.001 ^a^	3.90 ± 2.26
Mean thickness of SMH, µm	474.6 ± 149.6	791.9 ± 482.0	955.4 ± 375.9	<0.001 ^a^	625.5 ± 357.7
Classification of lesions (%)				0.028 ^b^	
CNV	15 (33.3)	12 (57.1)	8 (72.7)		35 (45.5)
PCV	30 (66.7)	9 (42.9)	3 (27.3)		42 (54.5)
Preoperative anti-VEGF treatment	2 (4.4)	3 (14.3)	5 (45.5)	0.005 ^b^	10 (13.0)
Lens status (%)				0.119 ^b^	
Phakic	40 (88.9)	14 (66.7)	8 (72.7)		62 (80.5)
Pseudophakic	5 (11.1)	7 (33.3)	3 (27.3)		15 (19.5)
Treatment modalities (%) ^b^				0.011 ^b^	
Intravitreal anti-VEGF monotherapy	23 (51.1)	6 (28.6)	0 (0.0)		29 (37.7)
Intravitreal anti-VEGF with C_3_F_8_ gas	12 (26.7)	9 (42.8)	4 (36.4)		25 (32.5)
PPV with subretinal tPA and SF_6_ gas	10 (22.2)	6 (28.6)	7 (63.6)		23 (29.8)

Values are presented as mean ± SD unless indicated otherwise; ^a^ *p*-value using analysis of variance or a Kruskal–Wallis test; ^b^ *p*-value using χ^2^ test or Fisher’s exact test; C_3_F_8_, perfluoropropane; CNV, choroidal neovascularization; ODD, optic disc diameter; PCV, polypoidal choroidal vasculopathy; PPV, pars plana vitrectomy; SF_6_, sulfahexafluoride; SMH, submacular hemorrhage; tPA, tissue plasminogen activator; VEGF, vascular endothelial growth factor.

**Table 2:** Functional and anatomical outcomes.

	Small-Sized SMH	Medium-Sized SMH	Large-Sized SMH	*p*-Value	Total
LogMAR BCVA					
Baseline (*n* = 77)	1.06 ± 0.70	1.5 ± 0.89	1.93 ± 0.84	0.015 ^a^	1.30 ± 0.83
1 month (*n* = 77)	0.83 ± 0.55	1.33 ± 0.83	2.45 ± 0.94	0.002 ^a^	1.19 ± 0.87
3 months (*n* = 64)	0.69 ± 0.65	1.26 ± 0.83	2.04 ± 1.05	0.018 ^a^	1.03 ± 0.95
6 months (*n* = 51)	0.59 ± 0.56	1.24 ± 0.90	1.84 ± 1.11	0.145 ^a^	1.00 ± 0.93
12 months (*n* = 41)	0.41 ± 0.45	1.14 ± 0.85	1.52 ± 0.89	0.139 ^a^	0.76 ± 0.83
Mean follow-up period, months	17.8 ± 19.4	17.7 ± 15.1	16.2 ± 12.9	0.963 ^a^	16.2 ± 17.4
Complete displacement or regression of SMH from the fovea (%)	37/45 (82.2)	13/21(61.9)	8/11(72.7)	0.200 ^b^	
Anti-VEGF monotherapy	18/23 (78.3)	2/6 (33.3)	–		20/29 (69.0)
Intravitreal anti-VEGF with C_3_F_8_ gas	9/12 (75.0)	6/9 (66.7)	1/4 (25.0)		16/25 (64.0)
PPV with a subretinal tPA and SF_6_ gas	10/10 (100.0)	5/6 (83.3)	6/7 (85.7)		21/23 (91.3)
Additional intravitreal anti-VEGF injections					
No. of eyes treated with anti-VEGF (%)	30 (66.7)	15 (71.4)	8 (72.7)	0.886 ^b^	53 (68.8)
No. of injections for 12 months	3.27 ± 2.06	2.60 ± 1.45	3.00 ± 1.85	0.526 ^a^	3.04 ± 1.83
Breakthrough hemorrhage (%)	3 (6.7)	1 (4.8)	4 (36.4)	0.009 ^b^	8 (1.04)
Recurrent SMH	0	1	0		1
Complications					
RPE rip	0	1	0		1
Macular hole	0	1	0		1
Retinal detachment	0	0	0		0

Values are presented as mean ± SD unless indicated otherwise; ^a^ *p*-values indicate the significance of difference among the subgroups using analysis of variance or a Kruskal–Wallis test; ^b^ *p*-values using χ^2^ test or Fisher’s exact test; BCVA, best-corrected visual acuity; C_3_F_8_, perfluoropropane; LogMAR, logarithm of the minimum angle of resolution; PPV, pars plana vitrectomy; RPE, retinal pigment epithelium; SF_6_, sulfahexafluoride; SMH, submacular hemorrhage; tPA, tissue plasminogen activator; VEGF, vascular endothelial growth factor.

**Table 3:** Subgroup analysis of treatment outcomes based on SMH size.

	Small-Sized SMH Group				Medium-Sized SMH Group				Large-Sized SMH Group		
	Anti-VEGF Monotherapy	C_3_F_8_/Anti-VEGF	PPV/tPA/SF_6_	*p*-Value	Anti-VEGF Monotherapy	C_3_F_8_/Anti-VEGF	PPV/tPA/SF_6_	*p*-Value	C_3_F_8_/Anti-VEGF	PPV/tPA/SF_6_	*p*-Value
	(*n* = 23)	(*n* = 12)	(*n* = 10)		(*n* = 6)	(*n* = 9)	(*n* = 6)		(*n* = 4)	(*n* = 7)	
Mean size of SMH, ODD	2.00 ± 0.80	2.56 ± 0.70	3.34 ± 0.59	0.001 ^a^	5.93 ± 2.00	4.73 ± 0.84	4.77 ± 0.39	0.148 ^a^	8.46 ± 2.23	7.03 ± 2.30	0.345 ^b^
Mean thickness of SMH, μm	431.6 ± 131.0	541.4 ± 194.7	493.3 ± 100.7	0.088 ^a^	669.6 ± 470.0	794.6 ± 420.3	910.0 ± 625.2	0.551 ^a^	1006.4 ± 259.6	921.4 ± 458.7	0.450 ^b^
Duration of SMH, days	21.4 ±39.1	8.3 ± 9.2	16.4 ± 27.3	0.370 ^a^	28.6 ± 35.8	4.7 ± 1.9	4.5 ± 3.4	0.067	17.8 ± 14.2	9.3 ± 10.2	0.214 ^b^
LogMAR visual acuity (No.)											
Baseline	0.95 ± 0.62 (23)	0.95 ± 0.59 (12)	1.41 ± 0.89 (10)	0.282 ^a^	1.47 ± 1.22 (6)	1.46 ± 0.93 (9)	1.61 ± 0.49 (6)	0.703 ^a^	1.96 ± 1.09 (4)	1.91 ± 0.76 (7)	0.923 ^b^
1 month	0.72 ± 0.57 (23)	0.91 ± 0.62 (12)	1.03 ± 0.31 (10)	0.036 ^a^	1.42 ± 1.18 (6)	1.23 ± 0.85 (9)	1.40 ± 0.39 (6)	0.756 ^a^	2.38 ± 1.03 (4)	2.50 ± 0.97 (7)	0.879 ^b^
3 months	0.49 ± 0.41 (20)	0.82 ± 0.77 (9)	0.99 ± 0.85 (9)	0.266 ^a^	1.51 ± 1.30 (5)	1.02 ± 0.66 (7)	1.35 ± 0.47 (5)	0.679 ^a^	2.42 ± 0.95 (4)	1.74 ± 1.13 (5)	0.421 ^b^
6 months	0.50 ± 0.45 (16)	0.73 ± 0.65 (6)	0.71 ± 0.79 (6)	0.850 ^a^	1.42 ± 1.39 (5)	1.17 ± 0.70 (6)	1.12 ± 0.54 (4)	0.779 ^a^	2.45 ± 0.63 (3)	1.60 ± 1.22 (5)	0.417 ^b^
12 months	0.21 ± 0.22 (10)	0.39 ± 0.41 (6)	0.83 ± 0.62 (5)	0.209 ^a^	1.47 ± 1.25 (4)	0.93 ± 0.75 (5)	1.08 ± 0.62 (4)	0.818 ^a^	2.45 ± 0.95 (3)	1.05 ± 0.58 (4)	0.064 ^b^
Mean follow-up period, months	16.3 ± 15.6	15.3 ± 19.9	24.1 ± 26.6	0.512 ^a^	20.3 ± 20.9	14.3 ± 13.0	20.2 ± 12.8	0.695 ^a^	18.0 ± 13.7	15.1 ± 13.4	0.744 ^b^

Values are presented as mean ± SD unless indicated otherwise; ^a^ *p*-values indicate the significance of difference among the subgroups using analysis of variance or a Kruskal–Wallis test; ^b^ *p*-values using a Mann–Whitney test; C_3_F_8_, perfluoropropane; LogMAR, logarithm of the minimum angle of resolution; ODD, optic disc diameter; PPV, pars plana vitrectomy; SF_6_, sulfahexafluoride; SMH, submacular hemorrhage; tPA, tissue plasminogen activator; VEGF, vascular endothelial growth factor.
